# Supplementary material for: Variant antigen diversity in Trypanosoma vivax is not driven by recombination
Source: Nat Commun. 2020 Feb 12;11:844. doi: 10.1038/s41467-020-14575-8 (PMC7015903; doi:10.1038/s41467-020-14575-8)
Supplement: Supplementary file 1 — Supplementary Information [file 41467_2020_14575_MOESM1_ESM.pdf]

Variant antigen diversity in *Trypanosoma vivax* is not driven by recombination

Silva Pereira et al.

Supplementary Information

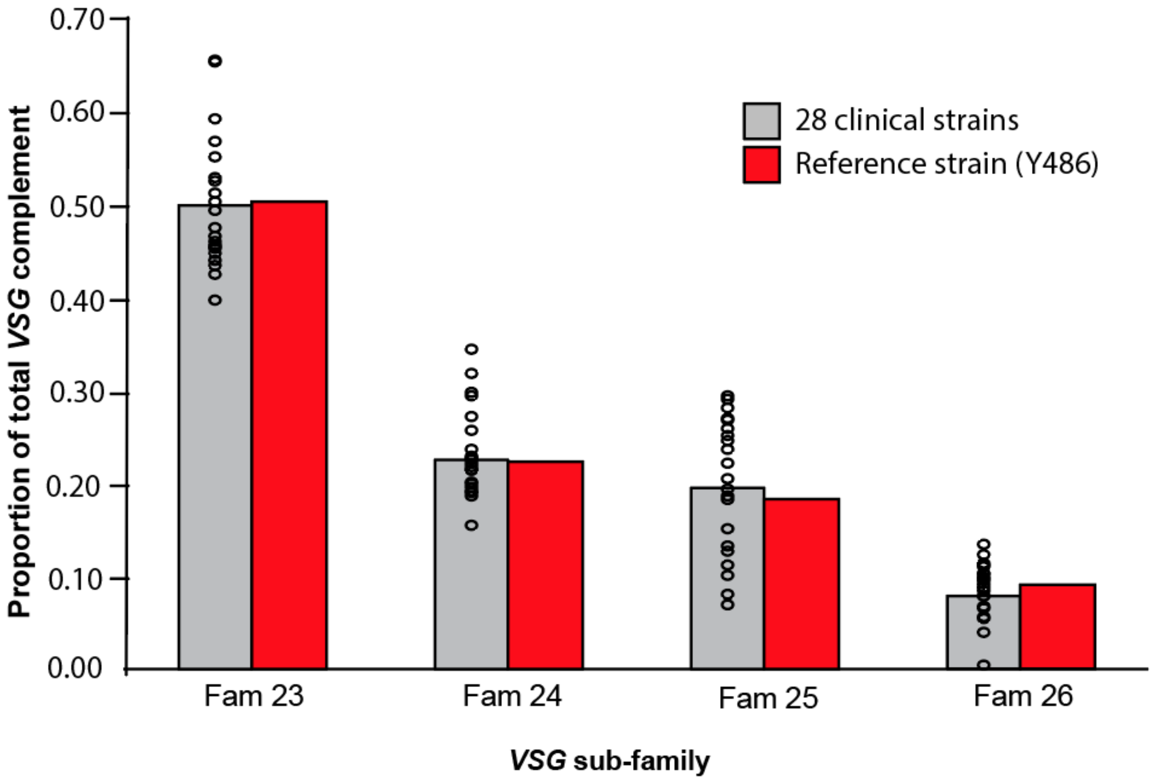

Supplementary figure 1. **Proportions of four conserved *VSG* sub-families (Fam23-26) to total *VSG* complement in 28 *T. vivax* clinical strains compared with the *T. vivax* Y486 reference strain.** Previously, we established Fam23-26 in *T. vivax* Y486 (Jackson et al. 2013). These were observed in all clinical strain genome sequences and in approximately the same relative proportions. This makes the sub-families unsuitable for discriminating among strains and therefore as a basis for variant antigen profiling, and requires the use of more variable taxa (i.e. phylotypes). Source data are provided as a Source Data file.

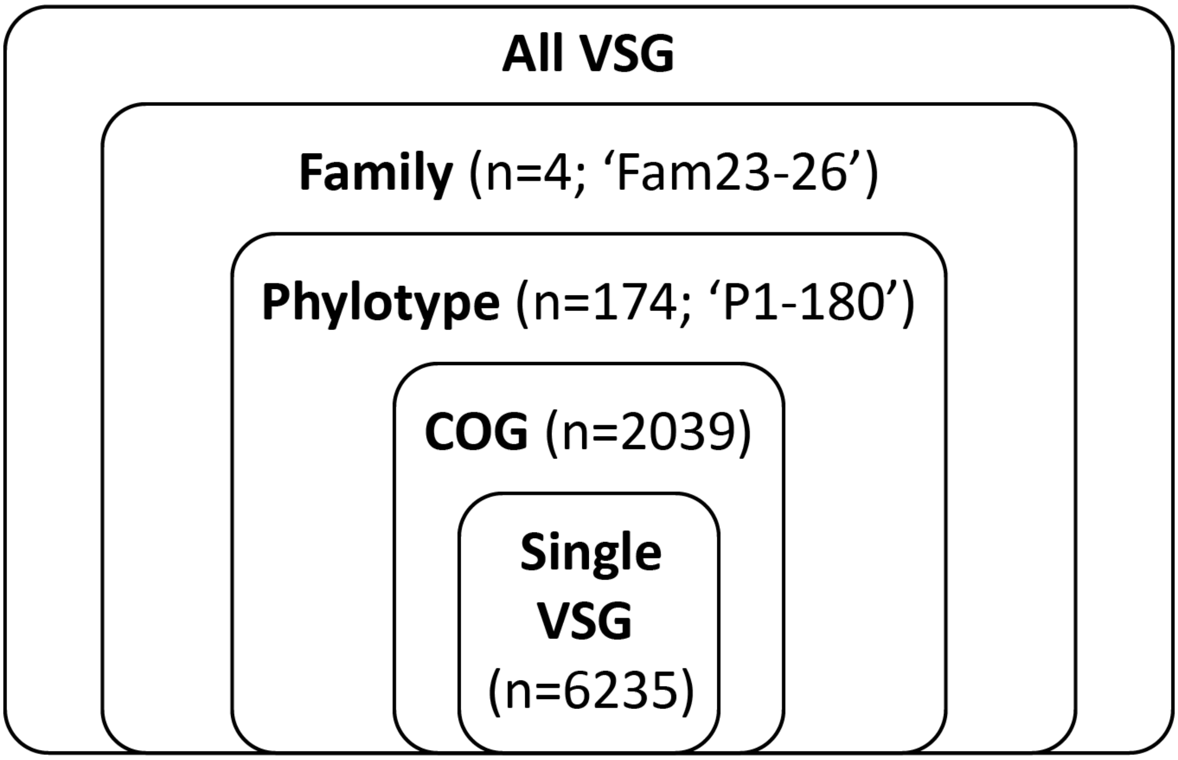

Supplementary figure 2. Classification of *T. vivax* VSG sequences in this study. This cartoon clarifies the nomenclature used to describe *T. vivax* VSG here. Taking all VSG-like sequences in all *T. vivax* genome sequences (i.e. 28 strains plus the Y486 reference), these can first be split into four **families** called Fam23-26 inclusive. These families were first established in our analysis of the Y486 sequence (Jackson et al. 2012, 2013). This study shows that Fam23-26 occur in approximately the same proportions in all strains and, therefore, cannot produce a discriminative variant antigen profile. Each family are further subdivided into 174 **phylotypes**, which we define here as monophyletic groups of VSG paralogs, displayed at least 70% average amino acid sequence identity across an alignment. Phylotypes vary by size between strains and have variable distribution; as such, they are suitable for variant antigen profiling. Note that phylotypes are numbered P1 to P180 but not inclusively, due to certain numbers being discontinued for historical reasons. Phylotypes are further subdivided into 2039 individual VSG genes, (or very closely related paralogs), present in multiple strains, which are called **clusters of orthologous genes (COGs).** We define these as monophyletic groups of VSG orthologs, displaying at least 90% average amino acid sequence identity across an alignment. Due to the incompleteness of the strain genomes in our study, the absence of individual COGs from a given repertoire cannot be interpreted as genuine absence in that strain (and for this reason, profiling is based on phylotypes).

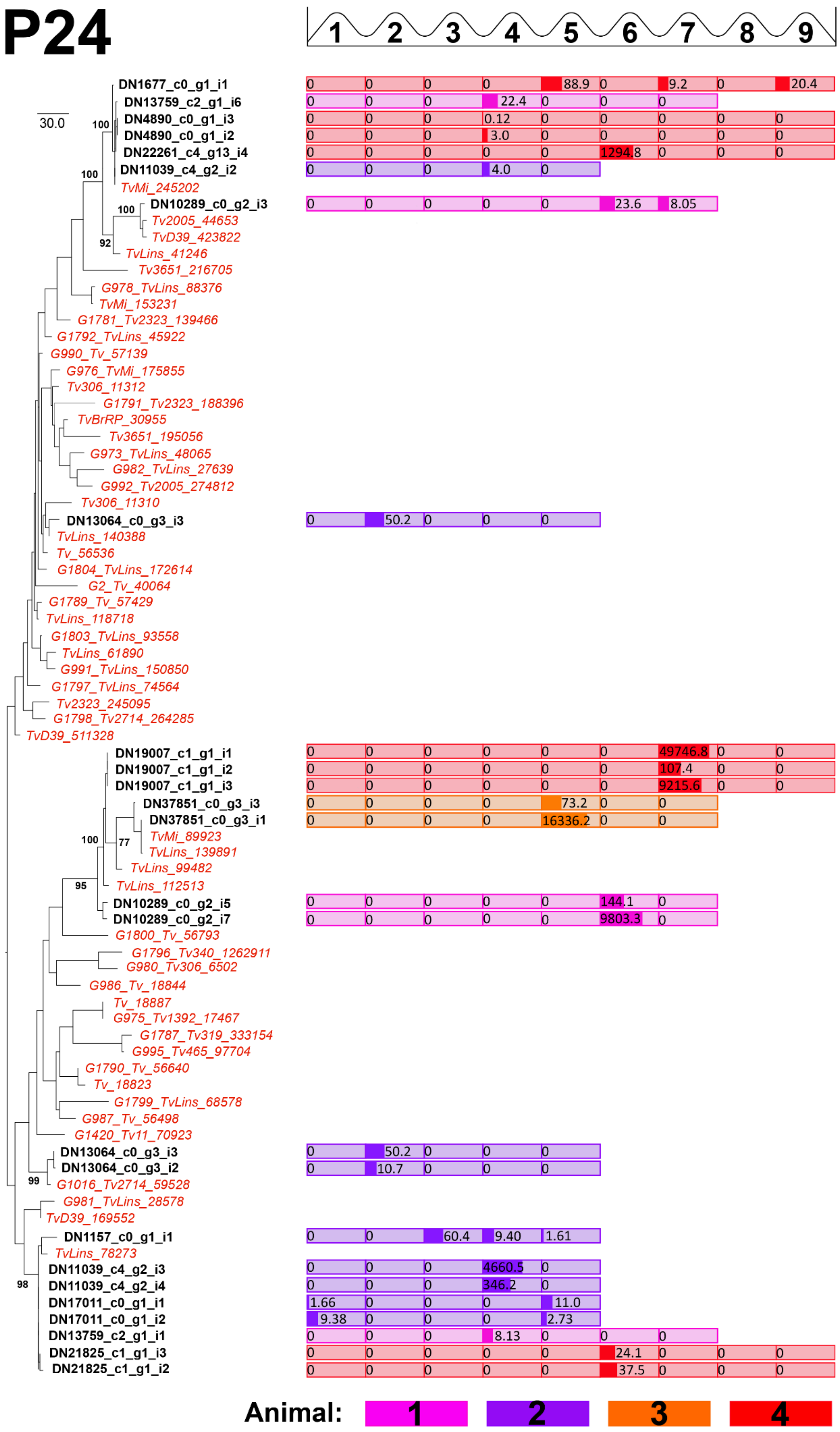

P24
1 2 3 4 5 6 7 8 9
30.0
100
100
100
92
77
100
95
99
98
DN1677_c0_g1_i1
DN13759_c2_g1_i6
DN4890_c0_g1_i3
DN4890_c0_g1_i2
DN22261_c4_g13_i4
DN11039_c4_g2_i2
TvMi_245202
DN10289_c0_g2_i3
Tv2005_44653
TvD39_423822
TvLins_41246
Tv3651_216705
G978_TvLins_88376
TvMi_153231
G1781_Tv2323_139466
G1792_TvLins_45922
G990_Tv_57139
G976_TvMi_175855
Tv306_11312
G1791_Tv2323_188396
TvBrRP_30955
Tv3651_195056
G973_TvLins_48065
G982_TvLins_27639
G992_Tv2005_274812
Tv306_11310
DN13064_c0_g3_i3
TvLins_140388
Tv_56536
G1804_TvLins_172614
G2_Tv_40064
G1789_Tv_57429
TvLins_118718
G1803_TvLins_93558
TvLins_61890
G991_TvLins_150850
G1797_TvLins_74564
Tv2323_245095
G1798_Tv2714_264285
TvD39_511328
DN19007_c1_g1_i1
DN19007_c1_g1_i2
DN19007_c1_g1_i3
DN37851_c0_g3_i3
DN37851_c0_g3_i1
TvMi_89923
TvLins_139891
TvLins_99482
TvLins_112513
DN10289_c0_g2_i5
DN10289_c0_g2_i7
G1800_Tv_56793
G1796_Tv340_1262911
G980_Tv306_6502
G986_Tv_18844
Tv_18887
G975_Tv1392_17467
G1787_Tv319_333154
G995_Tv465_97704
G1790_Tv_56640
Tv_18823
G1799_TvLins_68578
G987_Tv_56498
G1420_Tv11_70923
DN13064_c0_g3_i3
DN13064_c0_g3_i2
G1016_Tv2714_59528
G981_TvLins_28578
TvD39_169552
DN1157_c0_g1_i1
TvLins_78273
DN11039_c4_g2_i3
DN11039_c4_g2_i4
DN17011_c0_g1_i1
DN17011_c0_g1_i2
DN13759_c2_g1_i1
DN21825_c1_g1_i3
DN21825_c1_g1_i2
0 0 0 0 88.9 0 9.2 0 20.4
0 0 0 22.4 0 0 0
0 0 0 0.12 0 0 0 0 0
0 0 0 3.0 0 0 0 0 0
0 0 0 0 0 1294.8 0 0 0
0 0 0 4.0 0
0 0 0 0 0 23.6 8.05
0 50.2 0 0 0
0 0 0 0 0 0 49746.8 0 0
0 0 0 0 0 0 107.4 0 0
0 0 0 0 0 0 9215.6 0 0
0 0 0 0 73.2 0 0
0 0 0 0 16336.2 0 0
0 0 0 0 0 144.1 0
0 0 0 0 0 9803.3 0
0 50.2 0 0 0
0 10.7 0 0 0
0 0 60.4 9.40 1.61
0 0 0 4660.5 0
0 0 0 346.2 0
1.66 0 0 0 11.0
9.38 0 0 0 2.73
0 0 0 8.13 0 0 0
0 0 0 0 0 24.1 0 0 0
0 0 0 0 0 37.5 0 0 0
Animal: 1 2 3 4

Supplementary figure 3. **Maximum likelihood phylogeny of Phylotype 24 genes from reference and strain genomes (COG type sequences, shown in red) as well as expressed *VSG* sequences from *T. vivax* Lins (N = 25; shown in black).** The tree was estimated from a 585 bp amino acid alignment using a GTR+Γ model in RAXML (Stamatakis 2014). Some gene sequences were removed because they were too short. Robustness values (100 non-parametric bootstraps) are shown beside selected internal nodes. Transcript abundance values (CPM) for each peak of parasitaemia are shown beside associated terminal nodes. These values are shaded by animal replicate.

P24 was observed at 15/29 peaks across four replicates and comprised an average of 2.33±1.3 transcripts per observation. Phylotypes were routinely observed to comprise multiple, distinct transcripts. P24 comprised a single transcript on 5/15 peaks when it was observed. The maximum number of unique P24 transcripts observed at a single peak was five (A3, peak 4).

Individual phylotypes were reproducibly expressed in different animals. P24 was unique among phylotypes by providing a superabundant, dominant *VSG* in all animals in late infection (see Figure 4). However, the specific transcripts implicated in different animals are not identical. They are sometimes very closely related (e.g. superabundant P24 transcripts in A1 (DN10289) and A4 (DN19007) share 94.9% nucleotide identity), but elsewhere they are not (e.g. the superabundant P24 transcripts in A2 (DN11039) and A3 (DN37851) are only 73.5% identical).

Typically, where a phylotype was observed in multiple animals, (irrespective of superabundance), the transcripts concerned were not identical. However, there are examples of identical transcripts expressed in multiple animals, for example, transcript DN13759_c2_g1_i1 in A1 and DN11039_c4_g2_i4 in A2, as well as DN11039_c4_g2_i2 in A2 and DN22261 in A4.

Phylotypes persisted across consecutive peaks in the same animal. P24 was observed at every peak in A2, being superabundant at peaks 4 and 5. Nine different transcripts contribute to this profile, and none of these persist throughout the experiment. However, the figure does include individual *VSG* persisting across peaks. For example, DN1677 was expressed at peaks 6, 8 and 10 in A4, DN1157 was expressed at peaks 4, 5 and 6 in A2, and DN10289_c0_g2_i3 was expressed at both peaks 6 and 7 in A1.

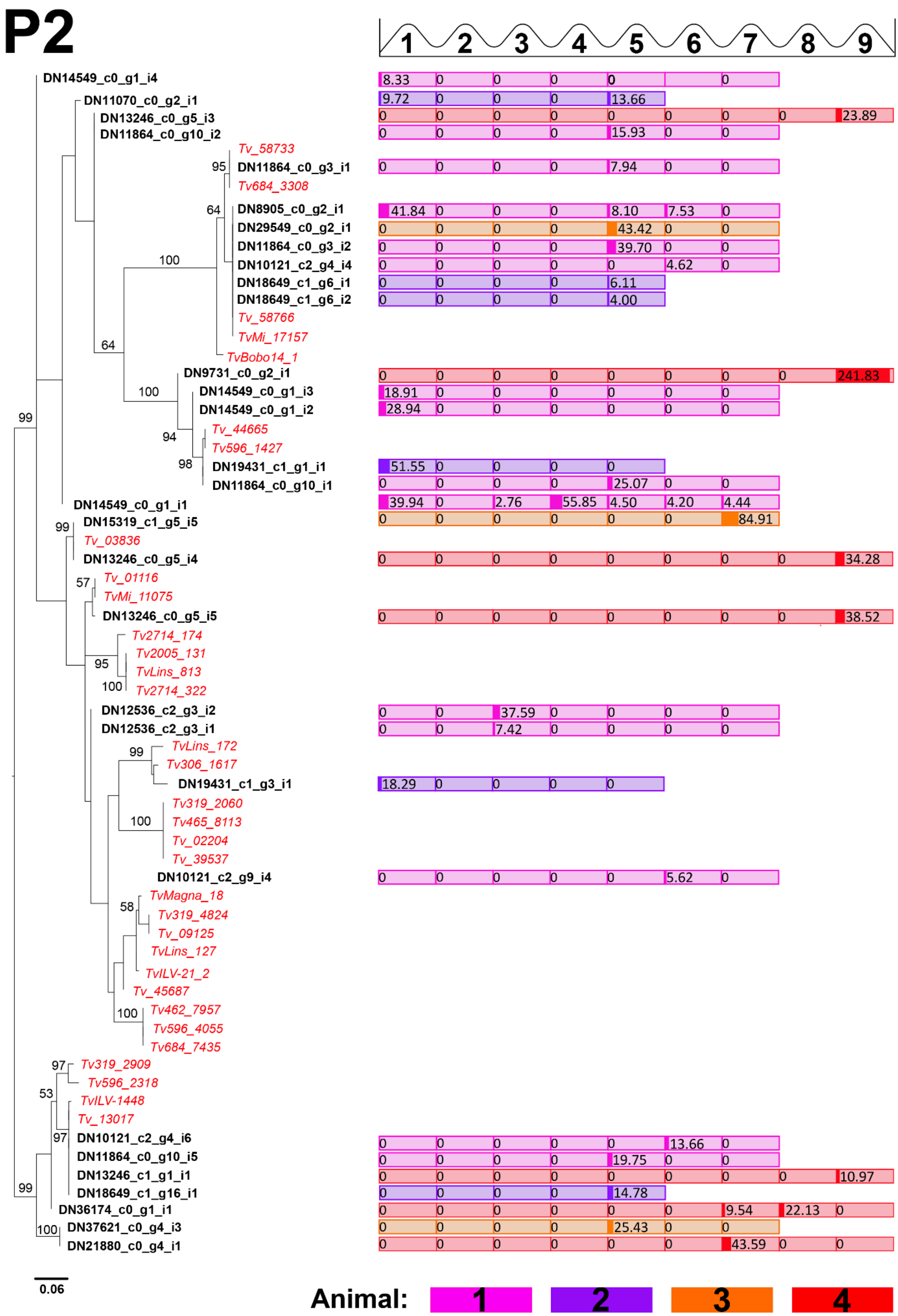

Sequence	1	2	3	4	5	6	7	8	9
DN14549_c0_g1_i4	8.33	0	0	0	0		0		
DN11070_c0_g2_i1	9.72	0	0	0	13.66				
DN13246_c0_g5_i3	0	0	0	0	0	0	0	0	23.89
DN11864_c0_g10_i2	0	0	0	0	15.93	0	0		
DN11864_c0_g3_i1	0	0	0	0	7.94	0	0		
DN8905_c0_g2_i1	41.84	0	0	0	8.10	7.53	0		
DN29549_c0_g2_i1	0	0	0	0	43.42	0	0		
DN11864_c0_g3_i2	0	0	0	0	39.70	0	0		
DN10121_c2_g4_i4	0	0	0	0	0	4.62	0		
DN18649_c1_g6_i1	0	0	0	0	6.11				
DN18649_c1_g6_i2	0	0	0	0	4.00				
DN9731_c0_g2_i1	0	0	0	0	0	0	0	0	241.83
DN14549_c0_g1_i3	18.91	0	0	0	0	0	0		
DN14549_c0_g1_i2	28.94	0	0	0	0	0	0		
DN19431_c1_g1_i1	51.55	0	0	0	0				
DN11864_c0_g10_i1	0	0	0	0	25.07	0	0		
DN14549_c0_g1_i1	39.94	0	2.76	55.85	4.50	4.20	4.44		
DN15319_c1_g5_i5	0	0	0	0	0	0	84.91		
DN13246_c0_g5_i4	0	0	0	0	0	0	0	0	34.28
DN13246_c0_g5_i5	0	0	0	0	0	0	0	0	38.52
DN12536_c2_g3_i2	0	0	37.59	0	0	0	0		
DN12536_c2_g3_i1	0	0	7.42	0	0	0	0		
DN19431_c1_g3_i1	18.29	0	0	0	0				
DN10121_c2_g9_i4	0	0	0	0	0	5.62	0		
DN10121_c2_g4_i6	0	0	0	0	0	13.66	0		
DN11864_c0_g10_i5	0	0	0	0	19.75	0	0		
DN13246_c1_g1_i1	0	0	0	0	0	0	0	0	10.97
DN18649_c1_g16_i1	0	0	0	0	14.78				
DN36174_c0_g1_i1	0	0	0	0	0	0	9.54	22.13	0
DN37621_c0_g4_i3	0	0	0	0	25.43	0	0		
DN21880_c0_g4_i1	0	0	0	0	0	0	43.59	0	0

Supplementary figure 4. **Maximum likelihood phylogeny of Phylotype 2 showing the relationships among constituent genes from reference and strain genomes (COG type sequences, shown in red) and expressed *VSG* sequences from *T. vivax* Lins (N = 31; shown in black).** The tree was estimated from a 228 bp alignment using a GTR+Γ model in RAXML (Stamatakis 2014). Robustness values (100 non-parametric bootstraps) are shown beside selected internal nodes. Beside terminal nodes representing expressed *VSG* are the transcript abundance values (CPM) for each peak of parasitaemia. These values are shaded by animal replicate.

P2 was observed at 13/29 peaks across four replicates and comprised an average of 3.08±1.9 transcripts per observation. Phylotypes were routinely observed to comprise multiple, distinct transcripts. P2 comprised a single transcript on 4/13 peaks when it was observed. The maximum number of unique P2 transcripts observed at a single peak was seven (A2, peak 5).

Individual phylotypes were reproducibly expressed in different animals; for example, P2 is superabundant in both A1 and A2 at peak 1. However, this implicates five different P2 transcripts in A1, but a single transcript in A2 (DN19431_c1_g3_i1), which was only seen in A2. While this is typical, the figure also provides examples of identical transcripts being expressed in different animals. For example, DN10121_c2_g4_i6 in A1 was identical to DN13246_c1_g1_i1 in A4 and DN18649_c1_g16_i1 in A2. Similarly, DN13246_c0_g5_i3 in A4 and DN11864_c0_g10_i2 in A4 are identical.

Phylotypes were observed to persist across consecutive peaks in the same animal. P2 provides a rare example in which the same transcript is expressed. DN14549_c0_g1_i1 was expressed throughout the experiment in A1 except at peak 2. While it was among the dominant VSG at peaks 1 and 4, it persisted at lower levels for the rest of the experiment, supplanted by other P2 transcripts in abundance. Elsewhere, individual transcripts re-emerged late after early expression, e.g. DN8905 was the dominant *VSG* in A1 at peak 1, and re-emerged at moderate levels during peaks 5 and 6, while DN11070 was expressed in A2 at peak 2 and re-emerged at peak 6 (as a low abundance transcript in both cases).

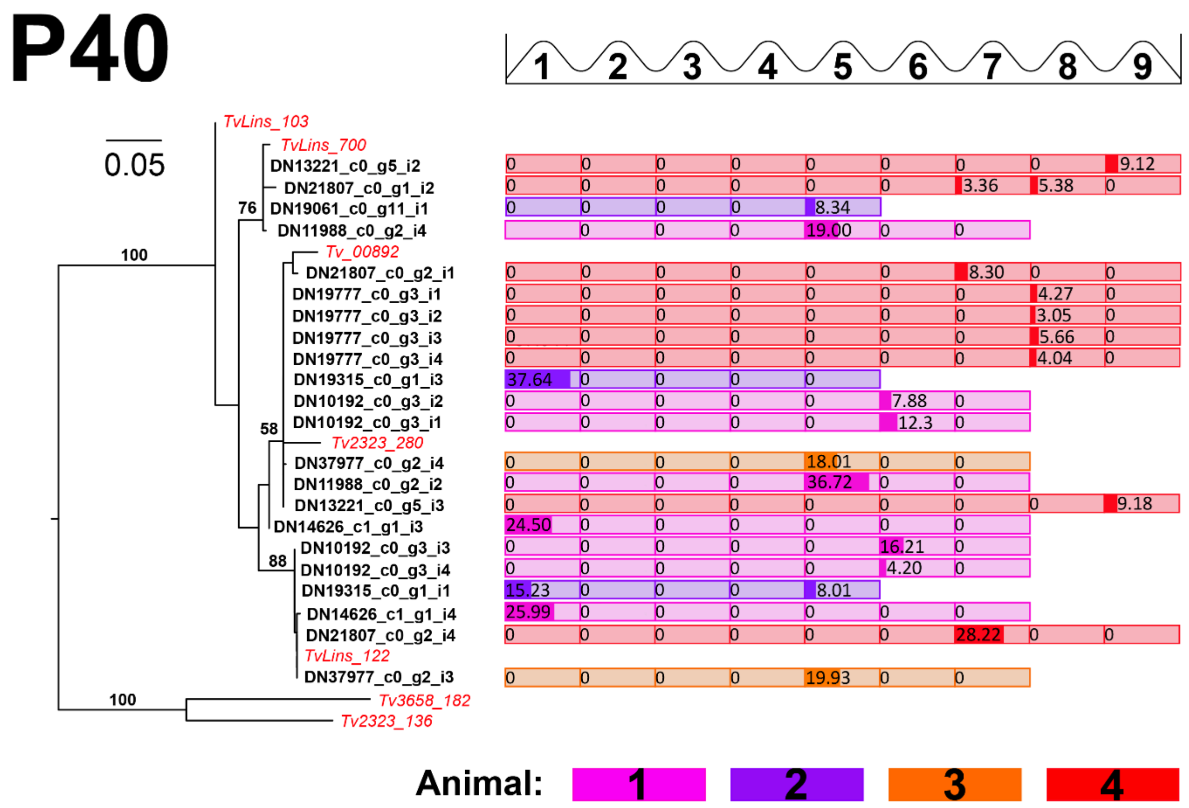

Supplementary Figure 5. Maximum likelihood phylogeny of Phylotype 40 showing the relationships among constituent genes from reference and strain genomes (COG type sequences, shown in red) and expressed VSG sequences from *T. vivax* Lins (shown in black). The tree was estimated from a 489 bp alignment using a GTR+Γ model in RAXML (Stamatakis 2014). Robustness values (100 non-parametric bootstraps) are shown beside selected internal nodes. Beside terminal nodes representing expressed *VSG* are the transcript abundance values (CPM) for each peak of parasitaemia. These values are shaded by replicate animal.

P40 was observed at 9/29 peaks across four replicates and comprised an average of 2.67±1.1 transcripts per observation. Phylotypes were routinely observed to comprise multiple, distinct transcripts. The maximum number of unique P40 transcripts observed at a single peak was five (A4, peak 9). P40 comprised a single transcript on 2/9 peaks when it was observed.

Individual phylotypes were reproducibly expressed in different animals, e.g. P40 is superabundant in both A1 and A2 at peak 1 (different transcripts), and co-dominant at peak 5 in A3 and A1. Again, the actual transcripts in the two animals are different. However, there are several examples of identical transcripts being observed as low abundance forms in multiple animals, e.g. DN19777 in A4 and DN10192 in A1 are identical to the superabundant DN19315_c0_g1_i3 in A2.

Phylotypes persisted across consecutive peaks in the same animal. P40 provides examples of this, e.g. across peaks 8-10 in A4, where it was superabundant at peak 8 and then present as multiple, low abundance transcripts thereafter. it was observed at every peak in A2, being superabundant at peaks 4 and 5. Typically when phylotypes persist or re-emerge, the actual transcripts concerned are distinct. However, transcript DN19315_c0_g1_i1 is expressed at peak 1 in A2, then again at peak 5, providing a rare example of the same transcript re-emerging during the experiment.

P143

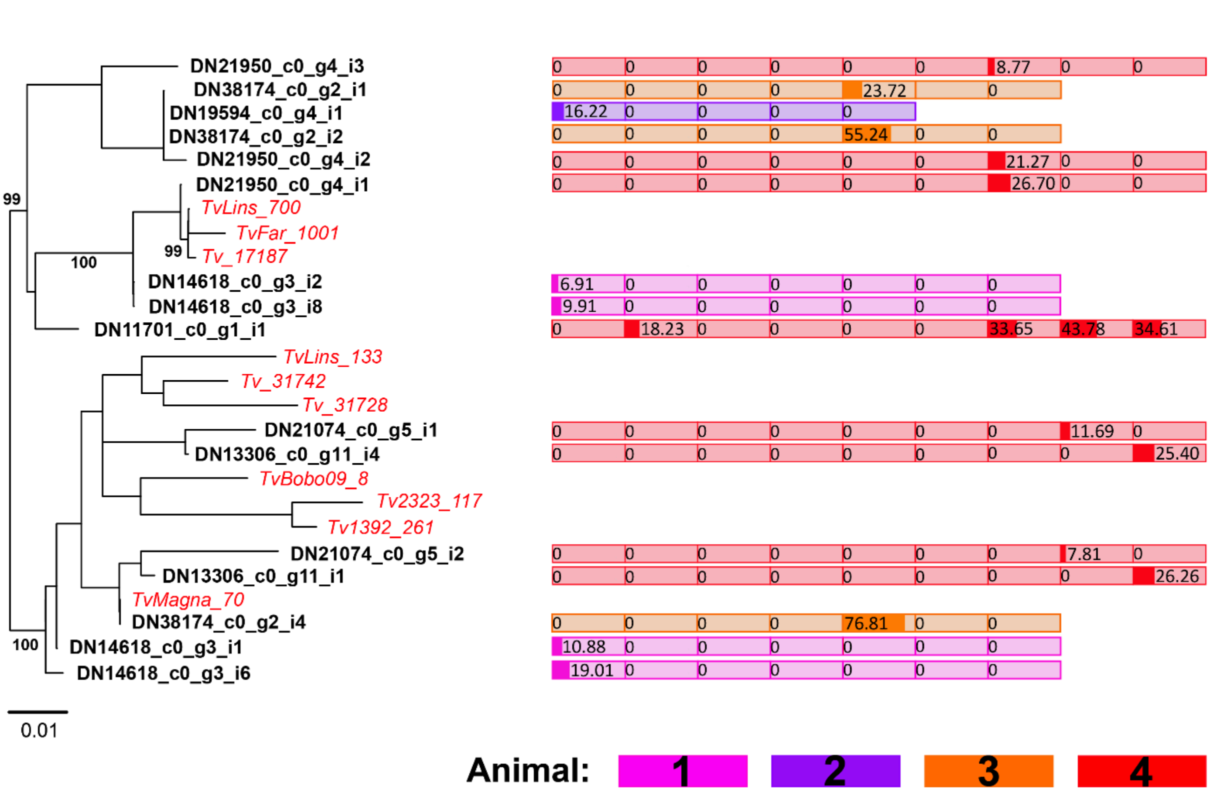

Supplementary Figure 6. Maximum likelihood phylogeny of Phylotype 143 showing the relationships among constituent genes from reference and strain genomes (COG type sequences, shown in red) and expressed VSG sequences from *T. vivax* Lins (shown in black). The tree was estimated from a 618 bp alignment using a GTR+Γ model in RAXML (Stamatakis 2014). Robustness values (100 non-parametric bootstraps) are shown beside selected internal nodes. Beside terminal nodes representing expressed VSG are the transcript abundance values (CPM) for each peak of parasitaemia. These values are shaded by animal replicate.

P143 was observed at 7/29 peaks across four replicates and comprised an average of 2.71±1.3 transcripts per observation. Phylotypes were routinely observed to comprise multiple, distinct transcripts. The maximum number of unique P143 transcripts observed at a single peak was four in A1 (peak 1) and A4 (peak 9). P40 comprised a single transcript on 2/7 peaks when it was observed.

Phylotypes were observed to persist across consecutive peaks in the same animal but, typically, the actual transcripts concerned are distinct. However, P143 provides an unusual example of a single transcript re-emerging and persisting. DN11701 was expressed at peak 3 in A4 (one of several superabundant VSG), and then again, at a much lower level, at peaks 8, 9 and 10.

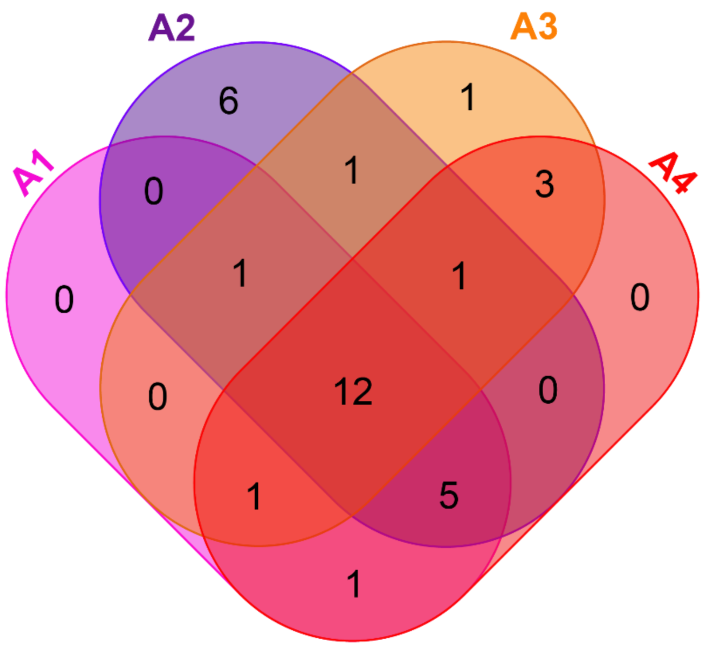

Phylotype	A1	A2	A3	A4
1	11	3	9	1
2	22	7	3	8
8	12	6	1	17
18	8	3	4	1
24	9	13	2	11
40	8	4	2	10
44	4	3	2	5
123	5	2	1	8
142	7	5	2	4
143	4	1	3	11
155	3	4	2	1
172	4	3	2	4
13	2	1	-	2
23	-	1	3	4
87	1	1	-	2
135	8	10	1	-
165	8	1	-	2
166	1	3	-	2
179	2	2	-	12
3	1	-	1	1
20	3	-	-	2
33	-	-	5	1
37	-	-	1	4
141	-	1	1	-
14	-	2	-	-
16	-	1	-	-
27	-	7	-	-
38	-	1	-	-
151	-	1	-	-
171	-	2	-	-
178	-	-	1	-

Supplementary Figure 7. A Venn diagram representing the phylotypes observed among expressed VSG transcripts in four animal replicates. Individual phylotype distribution is tabulated alongside.

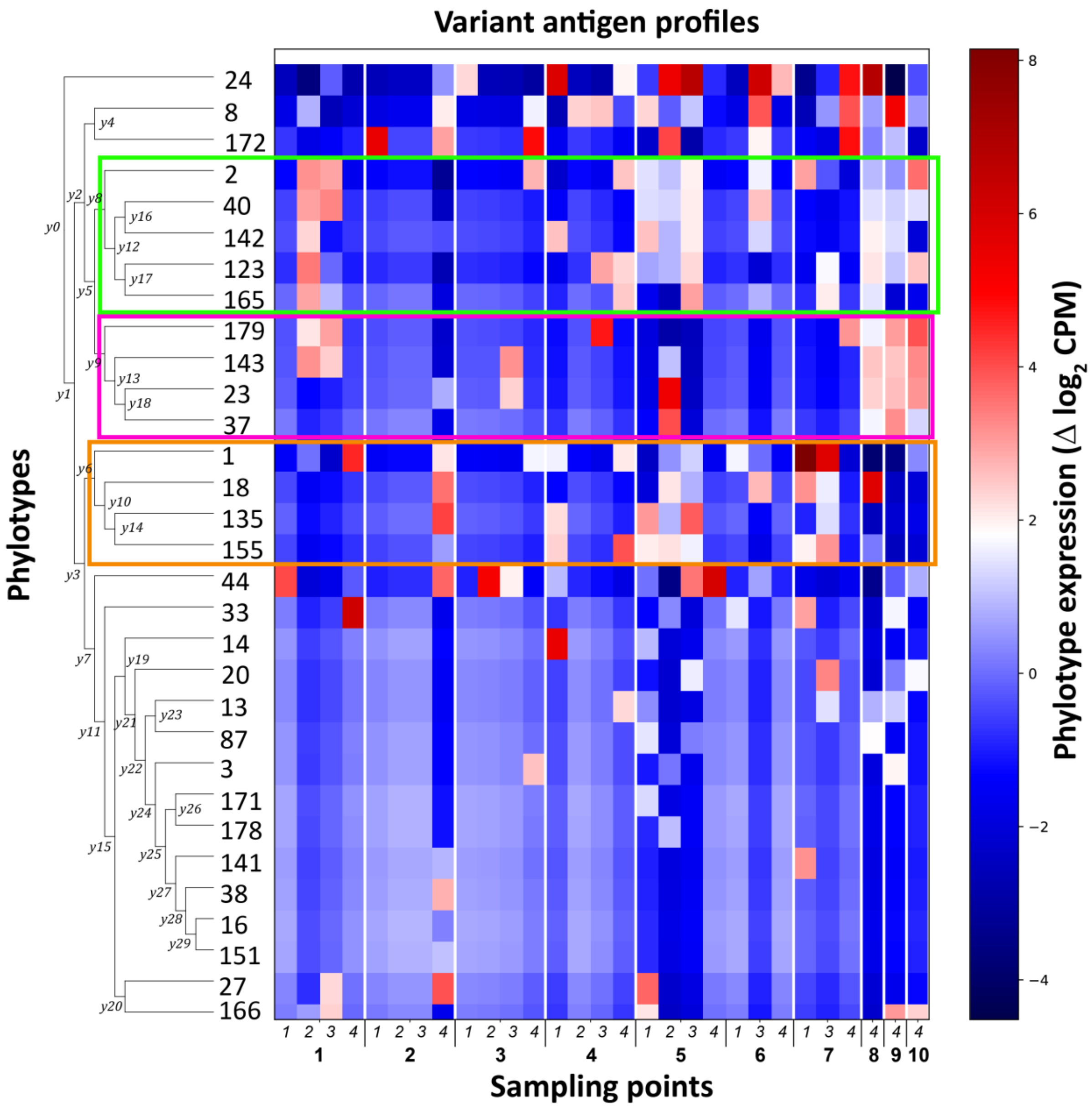

Supplementary Figure 8. **A Gneiss (balance) analysis of phylotype expression across each animal (Morton et al. 2017).** The heat map shows phylotype expression related to experimental time course. Cells in the map represent single sampling points from a given animal and peak of parasitaemia (x axis). On the x axis, animal number is shown in italics, and peaks of parasitaemia are numbered in bold. Cell shading represents changes in the combined transcript abundance from one peak to the next; red reflects increases in expression, blue represents decreases. Rows represent individual phylotypes; on the y-axis, these are arranged according to a dendrogram that clusters phylotypes based on Euclidean distances between phylotype profiles.

Each node of the dendrogram corresponds to a balance with each tip corresponding to a VSG phylotype. Significant differences in log ratio of time points were found in balances y1 (T8: $\beta = -7.1$, $p = 0.04$; T9: $\beta = -6.6$, $p = 0.05$) and y3 (T5: $\beta = -3.4$, $p = 0.05$; T7: $\beta = -4.8$, $p = 0.01$; T8: $\beta = -6.3$, $p = 0.03$), indicating that the repertoire of expressed phylotypes changed significantly at these points.

However, there were no significant differences in balances y7, y11 and y15 suggesting that the log abundances of phylotypes expressed then were not significantly different between time points.

The figure shows a modest reproducibility across animals in the identity and order of phylotypes, but substantial variation among replicates in expression timing. Bounded by a green line are examples of phylotypes (P2, P40, P142 and P143) expressed early (i.e. peak 1/2) in A2 and A3, that appear later at in A1-3 (peak 5/6), and even later in A4. A different set of phylotypes (P1, P18, P135 and P155) bounded by an orange line were expressed late (peak 5/7) in A1-3, but earlier in A4 (peak 2). In A4, where uniquely we have peaks 8-10, another set of phylotypes bounded by a pink line was expressed late (P179, P143, P23). Note that the phylotypes within these co-expressed groupings are often closely related. Source data are provided as a Source Data file.

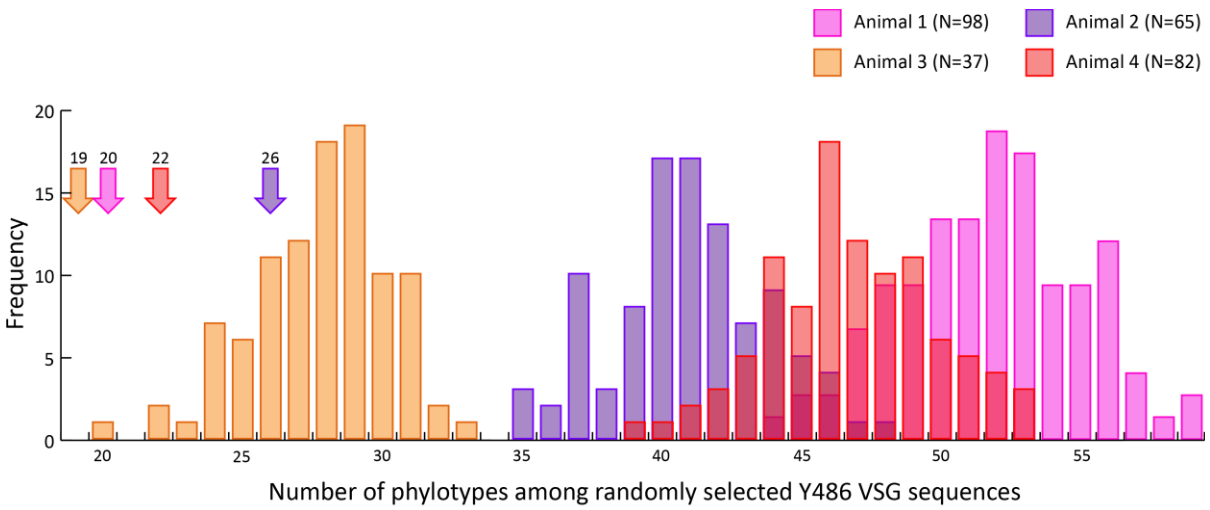

Supplementary figure 9. **Frequency distributions of the number of VSG phylotypes observed in simulated VSG repertoires.** VSG were selected at random from the Y486 repertoire to match the size of the observed repertoire and assigned to their phylotypes. This was repeated for each of four animal replicates. The observed number of phylotypes for each animal is indicated by arrows and is smaller than the simulated repertoires in each case. Furthermore, in each case the observed number lies outside of two standard deviations from the mean of the simulated repertoires. A1 expressed 20 phylotypes among 98 transcripts, simulated repertoires of the same size included mean average of 51.8 phylotypes (2xSD lower bound = 45.4). A2 expressed 26 phylotypes among 65 transcripts, simulated repertoires of the same size included mean average of 40.1 phylotypes (2xSD lower bound = 35.3). A3 expressed 19 phylotypes among 37 transcripts, simulated repertoires of the same size included mean average of 27.7 phylotypes (2xSD lower bound = 22.9). A4 expressed 22 phylotypes among 82 transcripts, simulated repertoires of the same size included mean average of 46.8 phylotypes (2xSD lower bound = 40.9). Source data are provided as a Source Data file.

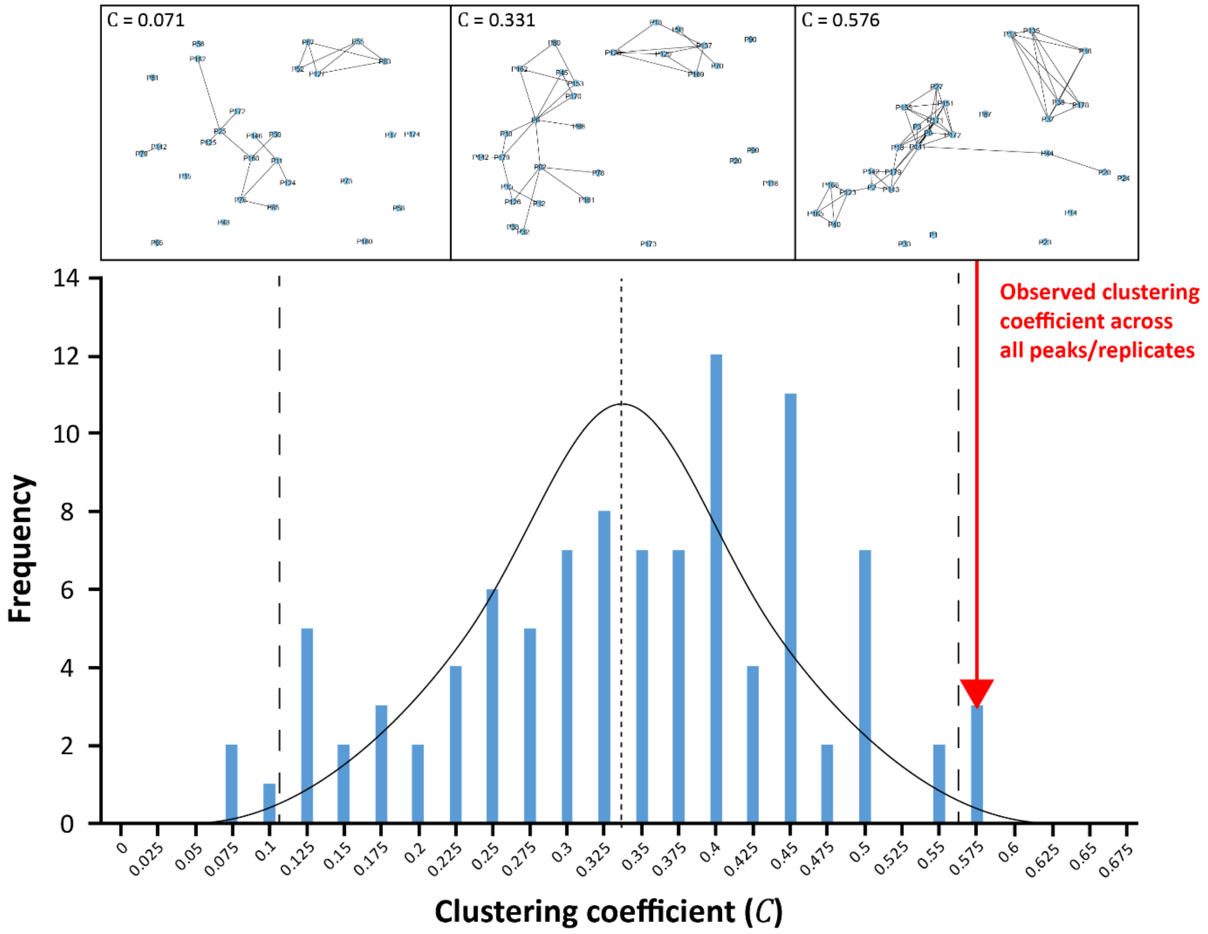

Supplementary Fig. 10. **Frequency distribution of clustering coefficients (*C*) for randomised sub-networks.** The phylotype networks shown in Figure 5 were analysed to compare the connectivity among 'expressed' nodes (i.e. those representing phylotypes that were expressed during experimental infections) with the connectivity expected by chance. *C* is a measure of the degree to which nodes in a graph tend to cluster together. The Network Analysis Tool in Cytoscape 3.7 was used to calculate the network average value for *C*, over all local clustering coefficients of all the vertices. Local clustering coefficients are computed as the proportion of connections among the immediate neighbours of a node that are realised compared with the number of all possible connections. Therefore, *C* is high where the nodes connected to a given node are themselves connected to each other. *C* was calculated for a subnetwork comprising all observed expressed nodes (N=31; red arrow). *C* was then calculated for 100 subnetworks of the same size where the nodes were selected by random number generator. The figure shows the distribution of *C*, which is approximately normal. Small and large dashed vertical lines represent the mean (0.333) and 2x standard deviations of the distribution respectively. The position of the observed value for *C* (0.576) exceeds twice the standard deviation of expected distribution (0.565), showing that the expressed nodes had a significantly higher value for *C* than would be expected by chance at P < 0.05. To illustrate, three subnetworks are shown above the distribution; a low-connectivity random subnetwork (left), a moderate-connectivity random network (centre) and the observed expressed node subnetwork (right). Source data are provided as a Source Data file.

Supplementary table 1. Matrix showing the proportion of VSG read-pairs from strain genomes mapping to unpaired locations in the reference genome by phylotype.

Strain VSG	Reference read														
Phylotypes	1	2	3	4	5	6	7	8	9	10	11	12	13	14	15
1	0.88	0	0.05	0	0	0	0	0	0	0	0	0	0.08	0	0
2	0	1	0	0	0	0	0	0	0	0	0	0	0	0	0
3	0.06	0.05	0.47	0	0	0	0	0	0	0	0	0	0	0	0.41
4	0	0	0	1	0	0	0	0	0	0	0	0	0	0	0
5	0	0	0	0.09	0.76	0.12	0.01	0.02	0	0	0	0	0	0	0
6	0	0	0	0.15	0.07	0.69	0.01	0.09	0	0	0	0	0	0	0
7	0	0	0	0	0	0	0.99	0	0	0	0	0	0	0	0
8	0	0	0	0.17	0.13	0.5	0	0.2	0	0	0	0	0	0	0
9	0	0	0	0	0	0	0	0	0.93	0	0.07	0	0	0	0
10	0	0	0	0	0	0	0	0	0	0.85	0	0	0	0	0.15
11	0	0	0	0	0	0	0	0	0	0	0.64	0.06	0.01	0.01	0.27
12	0	0	0	0	0	0	0	0	0	0	0.15	0.85	0	0	0
13	0	0	0	0	0	0	0	0	0	0	0.02	0.01	0.96	0	0.01
14	0	0	0	0	0	0	0	0	0	0	0.21	0	0	0.69	0.1
15	0	0.01	0.08	0	0	0	0	0	0	0	0.16	0	0.02	0	0.74

Note: Previously, we suggested that genetic exchange between T. congolense VSG phylotypes may be precluded by their distinct structures [Silva Pereira et al. 2018], and that this may effectively limit recombination rate [Jackson et al. 2012]. In this study, we found that T. brucei multi-coupled VSG have significantly higher TMRCA variance than T. congolense VSG ($p < 0.001$), indicating that the former may have a higher recombination rate. To test this, we mapped VSG sequence reads from 48 T. congolense strains to the IL3000 reference genome, and examined how the mapped loci related to 15 T. congolense phylotypes. After identifying read-pairs that became split after mapping, we calculated the proportion of these that split between two members of the same phylotype, as opposed to distinct phylotypes. Supplementary table 3 shows that split read-pairs remain largely mapped to the same phylotype, confirming an effective barrier to recombination between VSG clades. In T. brucei, all VSG share a homologous C-terminal domain that facilitates crossing-over, and no such barrier to mosaics has been found. Therefore, we believe that a difference in overall recombination rate between T. brucei and T. congolense is due indirectly to structural differences in their VSG genes.

References

Jackson, A.P., Berry, A., Aslett, M., Allison, H.C., Burton, P., Vavrova-Anderson, J., et al. Antigenic diversity is generated by distinct evolutionary mechanisms in African trypanosome species. Proc Natl Acad Sci U S A. **109** (9): 3416-21 (2012).

Jackson, A.P., Allison, H.C., Barry, J.D., Field, M.C., Hertz-Fowler, C., Berriman, M. A cell-surface phylome for African trypanosomes. *PLoS Negl Trop Dis*. **7** (3): e2121 (2013).

Morton, J.T., Sanders, J., Quinn, R.A., McDonald, D., Gonzalez, A., Vázquez-Baeza, Y., et al. Balance Trees Reveal Microbial Niche Differentiation. *mSystems* **12** (1): e00162-16 (2017).

Stamatakis, A. RAxML version 8: a tool for phylogenetic analysis and post-analysis of large phylogenies. *Bioinformatics* **30** (9): 1312-3 (2014).
