# Supplementary material for: Variant antigen diversity in Trypanosoma vivax is not driven by recombination
Source: Nat Commun. 2020 Feb 12;11:844. doi: 10.1038/s41467-020-14575-8 (PMC7015903; doi:10.1038/s41467-020-14575-8)
Supplement: Supplementary file 3 — Reporting Summary [file 41467_2020_14575_MOESM3_ESM.pdf]

nature research

Corresponding author(s): Dr Andrew P. Jackson (NCOMMS-19-29250A)

Last updated by author(s): Nov 27, 2019

Reporting Summary

Nature Research wishes to improve the reproducibility of the work that we publish. This form provides structure for consistency and transparency in reporting. For further information on Nature Research policies, seeAuthors & Referees and theEditorial Policy Checklist .

Statistics

For all statistical analyses, confirm that the following items are present in the figure legend, table legend, main text, or Methods section.

n/a	Confirmed	
☐	☒	The exact sample size (*n*) for each experimental group/condition, given as a discrete number and unit of measurement
☐	☒	A statement on whether measurements were taken from distinct samples or whether the same sample was measured repeatedly
☐	☒	The statistical test(s) used AND whether they are one- or two-sided<br>*Only common tests should be described solely by name; describe more complex techniques in the Methods section.*
☒	☐	A description of all covariates tested
☒	☐	A description of any assumptions or corrections, such as tests of normality and adjustment for multiple comparisons
☐	☒	A full description of the statistical parameters including central tendency (e.g. means) or other basic estimates (e.g. regression coefficient) AND variation (e.g. standard deviation) or associated estimates of uncertainty (e.g. confidence intervals)
☐	☒	For null hypothesis testing, the test statistic (e.g. *F*, *t*, *r*) with confidence intervals, effect sizes, degrees of freedom and *P* value noted<br>*Give P values as exact values whenever suitable.*
☒	☐	For Bayesian analysis, information on the choice of priors and Markov chain Monte Carlo settings
☒	☐	For hierarchical and complex designs, identification of the appropriate level for tests and full reporting of outcomes
☒	☐	Estimates of effect sizes (e.g. Cohen's *d*, Pearson's *r*), indicating how they were calculated

Our web collection on statistics for biologists contains articles on many of the points above.

Software and code

Policy information about availability of computer code

Data collection	Velvet (v1.2.10)<br>Orthofinder<br>Clustalx (v2.1)<br>Bowtie2 (v2.3.4)<br>BWA mem<br>SAMtools (v1.9)<br>Picard<br>Genome Analysis Toolkit (v3.8-0)<br>VCFtools (v0.1.14)<br>PHYML (v3.0)<br>Trinity (v2.8.6)<br>kallisto (v0.45.0)<br>BLAST+ (v2.8.0)

Data analysis	Bowtie2 (v2.3.4)<br>NetRecodon (v6.0.0)<br>Muscle (v3.8.31)<br>PhiPack<br>BEDtools (v2.27.0)<br>ACG<br>MEGA (v10.0.5)<br>GARD

For manuscripts utilizing custom algorithms or software that are central to the research but not yet described in published literature, software must be made available to editors/reviewers. We strongly encourage code deposition in a community repository (e.g. GitHub). See the Nature Research guidelines for submitting code & software for further information.

Data

Policy information about availability of data

All manuscripts must include a data availability statement. This statement should provide the following information, where applicable:

- Accession codes, unique identifiers, or web links for publicly available datasets
- A list of figures that have associated raw data
- A description of any restrictions on data availability

All DNA and RNA sequence data are provided without restriction via the European Nucleotide Archive, accession number PRJNA486085.

Field-specific reporting

Please select the one below that is the best fit for your research. If you are not sure, read the appropriate sections before making your selection.

[x] Life sciences [ ] Behavioural & social sciences [ ] Ecological, evolutionary & environmental sciences

For a reference copy of the document with all sections, see nature.com/documents/nr-reporting-summary-flat.pdf

Life sciences study design

All studies must disclose on these points even when the disclosure is negative.

Sample size	The sample size for genome sequencing (i.e. 28) was determined by availability of historical clinical stabilates, but efforts were made to sample across the ideal range (i.e. total species distribution). The sample size for experimental infections (i.e. 4) is a conventional minimum replication required to assess reproducibility in transcript abundance estimates.
Data exclusions	No data were excluded from the analyses.
Replication	We explicitly discuss the extent to which patterns in genomic and transcriptomic VSG repertoires are reproducible. The extent of this varies for biological reasons. To the extent that all genomic and transcriptomic replicates supported a common conclusion, there were no failed attempts to reproduce the results.
Randomization	This is not relevant to our study because we did not manipulate the samples for experimental effect.
Blinding	This is not relevant to our study because we did not manipulate the samples for experimental effect.

Reporting for specific materials, systems and methods

We require information from authors about some types of materials, experimental systems and methods used in many studies. Here, indicate whether each material, system or method listed is relevant to your study. If you are not sure if a list item applies to your research, read the appropriate section before selecting a response.

Materials & experimental systems

n/a	Involved in the study	
[x]	[ ]	Antibodies
[x]	[ ]	Eukaryotic cell lines
[x]	[ ]	Palaeontology
[ ]	[x]	Animals and other organisms
[x]	[ ]	Human research participants
[ ]	[x]	Clinical data

Methods

n/a	Involved in the study	
[x]	[ ]	ChIP-seq
[x]	[ ]	Flow cytometry
[x]	[ ]	MRI-based neuroimaging

Animals and other organisms

Policy information about studies involving animals; ARRIVE guidelines recommended for reporting animal research

Laboratory animals	Four male Saanen goats of 4 to 8 months of age
Wild animals	The study did not involve wild animals
Field-collected samples	This study did not involve samples collected from the field
Ethics oversight	This study was conducted in accordance with the guidelines of the Brazilian College of Animal Experimentation (CONCEA), following the Brazilian law for "Procedures for the Scientific Use of Animals" (11.794/ 2008 and decree 6.899/2009). Ethical approval was obtained from the Ethical Committee to the Use of Animals (CEUA) of the Veterinary and Agrarian Sciences Faculty (FCAV) of the State University of São Paulo (Jaboticabal campus) (São Paulo, Brazil) (protocol no. 001494/18, issued on 08/02/2018). The study was also approved by the Animal Welfare and Ethical Review Body (AWERB) of the University of Liverpool (AWC0103).

Note that full information on the approval of the study protocol must also be provided in the manuscript.

Clinical data

Policy information about clinical studies

All manuscripts should comply with the ICMJEguidelines for publication of clinical research and a completedCONSORT checklist must be included with all submissions.

Clinical trial registration	N/A
Study protocol	N/A
Data collection	N/A
Outcomes	N/A
