# Supplementary material for: Variant antigen diversity in Trypanosoma vivax is not driven by recombination
Source: Nat Commun. 2020 Feb 12;11:844. doi: 10.1038/s41467-020-14575-8 (PMC7015903; doi:10.1038/s41467-020-14575-8)
Supplement: Supplementary file 4 — Description of Additional Supplementary Files [file 41467_2020_14575_MOESM4_ESM.pdf]

Description of Additional Supplementary Files

File Name: Supplementary Data 1
Description: Names and details of isolation for *Trypanosoma vivax* clinical strains used in this study and descriptive statistics for their genome sequences.

File Name: Supplementary Data 2
Description: Index of VSG COGs and phylotypes, showing the membership of each (by individual 'type sequences') and the distribution of each across strains and locations.

File Name: Supplementary Data 3
Description: Descriptions of *T. vivax* transcriptomes created from blood samples extracted at peak parasitaemia during experimental infections.

File Name: Supplementary Data 4
Description: Names and details of isolation for African trypanosome parasite strains of which genome sequences were used in this study.
